# The mito-DAMP cardiolipin blocks IL-10 production causing persistent inflammation during bacterial pneumonia

**DOI:** 10.1038/ncomms13944

**Published:** 2017-01-11

**Authors:** Krishnendu Chakraborty, Mahesh Raundhal, Bill B. Chen, Christina Morse, Yulia Y. Tyurina, Anupriya Khare, Timothy B. Oriss, Rachael Huff, Janet S. Lee, Claudette M. St. Croix, Simon Watkins, Rama K. Mallampalli, Valerian E. Kagan, Anuradha Ray, Prabir Ray

**Affiliations:** 1 Pulmonary, Allergy and Critical Care Medicine, Department of Medicine, University of Pittsburgh School of Medicine, 3459 Fifth Avenue, Pittsburgh, Pennsylvania 15213, USA; 2 Department of Immunology, 200 Lothrop St, University of Pittsburgh School of Medicine, E1040 BSTWR, Pittsburgh, Pennsylvania 15261, USA; 3 Center for Free Radical and Antioxidant Health, Department of Environmental and Occupational Health, University of Pittsburgh School of Medicine, Bridgeside Point, 100 Technology Drive, Suite 350, Pittsburgh, Pennsylvania 15219, USA; 4 Center for Biologic Imaging, University of Pittsburgh School of Medicine, 3500 Terrace St, Pittsburgh, Pennsylvania 15261, USA

## Abstract

Bacterial pneumonia is a significant healthcare burden worldwide. Failure to resolve inflammation after infection precipitates lung injury and an increase in morbidity and mortality. Gram-negative bacteria are common in pneumonia and increased levels of the mito-damage-associated molecular pattern (DAMP) cardiolipin can be detected in the lungs. Here we show that mice infected with *Klebsiella pneumoniae* develop lung injury with accumulation of cardiolipin. Cardiolipin inhibits resolution of inflammation by suppressing production of anti-inflammatory IL-10 by lung CD11b^+^Ly6G^int^Ly6C^lo^F4/80^+^ cells. Cardiolipin induces PPARγ SUMOylation, which causes recruitment of a repressive NCOR/HDAC3 complex to the IL-10 promoter, but not the TNF promoter, thereby tipping the balance towards inflammation rather than resolution. Inhibition of HDAC activity by sodium butyrate enhances recruitment of acetylated histone 3 to the IL-10 promoter and increases the concentration of IL-10 in the lungs. These findings identify a mechanism of persistent inflammation during pneumonia and indicate the potential of HDAC inhibition as a therapy.

## Abstract (web summary)

Non-resolving bacterial pneumonia results in lung tissue damage owing to overactive inflammation. Here the authors show that the mitochondrial DAMP cardiolipin contributes to persistent inflammation by SUMOylating PPARγ, which promotes binding of the corepressor NCOR/HDAC3 complex to the IL-10 promoter.

Bacterial infection of the lung induces an inflammatory response, which is a natural useful response to eliminate the invading pathogen. However, unless terminated quickly, this inflammatory response can also precipitate pneumonia, which fails to resolve in some cases, resulting in a condition termed non-resolving pneumonia, which can cause acute lung injury^1^,^2^. Bacterial pneumonia continues to be a leading cause of morbidity and mortality worldwide^3^. Therefore, one of the pressing questions in medicine is how excess inflammation can be turned off. Resolution of inflammation is no longer considered a passive process but one that involves timely elaboration of different mediators that helps to terminate the inflammatory response and restore tissue homeostasis^4^,^5^,^6^.

Alveolar macrophages and neutrophils are the major cell types that phagocytose and kill internalized lung bacteria^7^,^8^, with alveolar macrophages responding immediately and subsequent infiltration of neutrophils in response to chemotactic stimuli^9^. Neutrophils generate noxious products including reactive oxygen species and proteases that are not only harmful to pathogens but also to the host^3^,^10^,^11^,^12^. Therefore, once the pathogen is cleared, the immediate goal of the host is to mount an appropriate anti-inflammatory response to limit further neutrophil recruitment and to clear apoptotic neutrophils in a process called neutrophil efferocytosis. The Gram-negative bacterium *Klebsiella pneumoniae* is frequently detected in hospital-acquired pneumonia, particularly in chronically ill patients, and is the third most commonly isolated organism from intensive care units in the United States^13^. We previously showed that CD11b^+^Ly6G^int^Ly6C^lo^F4/80^+^ cells characterized as lung myeloid-derived suppressor cells (MDSCs)^14^,^15^ have an important function in neutrophil efferocytosis after infection with *K. pneumoniae*^16^.

High concentrations of the phospholipid cardiolipin have been detected in the lung fluid of patients with persistent pneumonia^17^. Cardiolipin is the principal lipid component of the inner mitochondrial membrane. Although cardiolipin is normally confined to mitochondria, during cell death it undergoes oxidation and is released into the extracellular milieu as a mitochondrial damage-associated molecular pattern (mito-DAMP)^18^,^19^,^20^. Here we show that cardiolipin induces SUMOylation of the nuclear receptor PPARγ at K107, which is distinct from the previously described SUMOylation at K395 induced by the PPARγ agonist rosiglitazone (rosi)^21^. Cardiolipin-induced SUMOylation inhibits interleukin (IL)-10 production, but does not interfere with tumour necrosis factor (TNF) production, the latter being inhibited by rosi-induced K395 SUMOylation. K107 SUMOylation in PPARγ causes recruitment of the repressive NCOR/HDAC3 complex to the IL-10 upstream regulatory region, but spares the TNF promoter. Exposure of MDSCs to extracellular cardiolipin causes accumulation of cyclic phosphatidic acid (cPA), which also induces PPARγ SUMOylation and recruitment of HDAC3 to the IL-10 promoter. Inhibition of HDAC activity in mice infected with *K. pneumonia* restores lung IL-10 levels and reduces TNF with gain in body weight, plus increases survival of the mice. Our study identifies cardiolipin as a mediator of non-resolving pneumonia via SUMOylation of PPARγ in which HDAC inhibition provides therapeutic benefit.

## Results

### Elevated oxidized cardiolipin level in infected lungs

**Figure 1:**
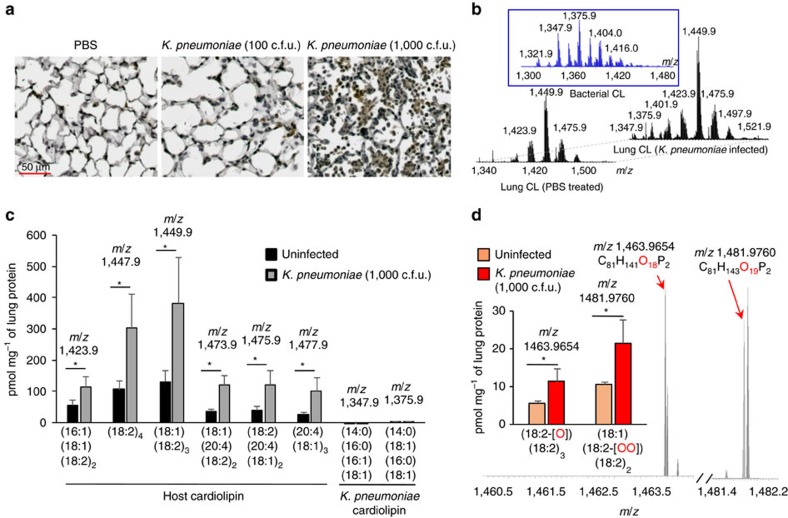
Increased level of cardiolipin in lungs of mice infected with *K. pneumoniae*. (**a**) C57BL/6J mice were either left uninfected or infected intratracheally (i.t.) with *K.pneumoniae* (100 or 1,000 c.f.u.). Day 3 post infection, lung tissue sections of mice were stained for oxidized phospholipids. (**b**) LC/MS spectra of host cardiolipin obtained from lungs of PBS-treated mice (left) and infected mice (right). Middle panel depicts LC/MS spectrum of bacterial cardiolipin. (**c**) Quantitation of major host and bacterial cardiolipin molecular species from spectral data shown in **b**. (**d**) LC–MS spectra of mono- (*m*/*z* 1,463.9654) and di- (*m*/*z* 1,481.9760) oxygenated cardiolipin molecular species and their quantitative assessments in the lung. Data shown are mean±s.d. and all data are representative of two independent experiments with *n*=3 mice per group. Significance was calculated using Student's unpaired two-tailed *t* test for comparisons involving two groups or one-way ANOVA with Tukey's *post hoc* test for multiple pairwise comparisons. *P<0.05. CL, cardiolipin.

We previously showed that mice infected with 100 colony-forming units (c.f.u.) of *K. pneumoniae* have a 100% survival rate while those infected with a higher dose (1,000 c.f.u.) show 50% survival^16^. When examined for the level of total oxidized phospholipids, a higher level was detected in the lung tissue of mice infected with 1,000 c.f.u. of *K. pneumoniae* as compared with that in mice infected with 100 c.f.u. (Fig. 1a). The level of extracellular host cardiolipin was found to be elevated in the lungs of mice infected with the higher bacterial dose (1,000 c.f.u.) when compared with uninfected controls (Fig. 1b,c). The level of bacterial cardiolipin was largely similar in the two groups. Moreover, the levels of oxidized forms (mono- and di-oxygenated) of the two most abundant C18:2-containing cardiolipin species were also higher in infected than in control samples (Fig. 1d).

**Figure 2:**
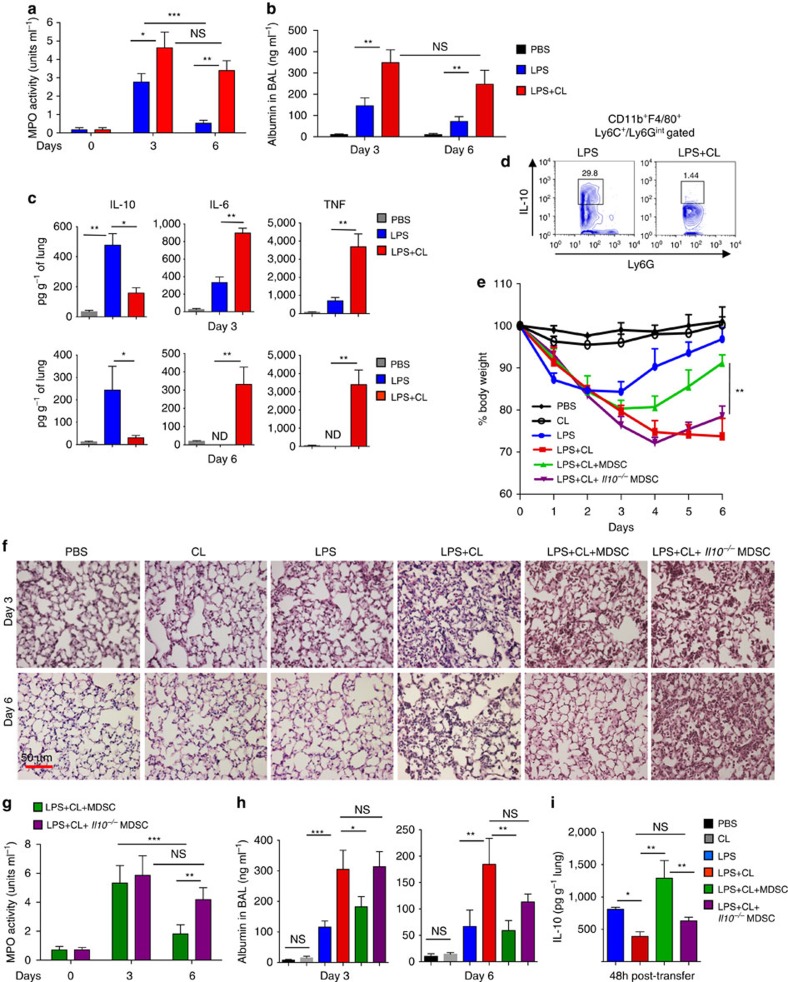
Induction of non-resolving lung inflammation with cardiolipin and low dose of LPS. (**a**,**b**) C57BL/6J mice were treated intratracheally (i.t.) with LPS (20 μg per mouse), either alone or in combination with cardiolipin (100 μg per mouse). Cardiolipin was administered 1 h post LPS instillation. PBS-treated mice were used as controls. On days 3 and 6 post-treatment, the mice were analysed for (**a**) myeloperoxidase activity (MPO) activity in BAL fluid, (**b**) BAL albumin levels, a measure of microvascular permeability and (**c**) levels of cytokines in lung homogenates measured using magnetic bead-based assay. (**d**) Mice were treated with LPS±cardiolipin for three consecutive days. Day 6 post-treatment, IL-10 expression in CD11b^+^Ly6C^lo^y6G^int^F4/80^+^ lung MDSCs was assayed by flow cytometry. Gating is shown in Supplementary Fig. 1. (**e**–**h**) Mice were treated with LPS either alone or in combination with cardiolipin. Twenty-four hour post-treatment, 1 × 10^6^ bone marrow-derived MDSCs from wild type or *Il10*^*−/−*^ mice were adoptively transferred i.t. into mice that had received both LPS and cardiolipin and the following parameters were studied day 3 and 6 post LPS±cardiolipin administration: (**e**) Change in body weight monitored over six days (presented as % change from initial body weight). (**f**) lung pathology by H&E staining of lung tissue sections, (**g**) MPO activity in BAL fluid and (**h**) BAL albumin level. (**i**) Level of IL-10 in lung homogenates measured 48 h post adoptive transfer. The experiment was initiated with the following numbers of mice: 3 for PBS, cardiolipin and LPS groups, 8 for LPS+cardiolipin, 7 for LPS+cardiolipin+MDSC (WT) and 8 for LPS+cardiolipin+MDSC (*Il10*^−/−^) groups. At the conclusion of the experiment on day 6, 3 animals in PBS, cardiolipin, LPS and LPS+cardiolipin groups, 5 mice in the LPS+cardiolipin+MDSC (WT) group and 3 mice in the LPS cardiolipin+MDSC (*Il10*^−/−^) were available for analysis. Statistical significance was calculated using one-way ANOVA or two-way ANOVA with Bonferroni's *post hoc* test was used for multiple pairwise comparisons. Data shown are mean±s.d. and data are representative of two independent experiments. *P≤0.05, **P≤0.01, ***P≤0.001; NS, not significant.

We recently reported that concomitant exposure of cells to cardiolipin and lipopolysaccharide (LPS) *in vitro* inhibits the production of pro-inflammatory cytokines such as TNF-α by the cells^22^. However, these studies did not address the impact of cardiolipin *in vivo* on already established inflammation when cardiolipin levels increase in the extracellular milieu (Fig. 1a). Also, given that an increased level of the mito-DAMP cardiolipin was found in the lungs of pneumonia subjects^17^, we hypothesized that cardiolipin in an inflammatory niche has the ability to incite a condition of non-resolving lung inflammation. To test our hypothesis, a low dose of bacterial LPS was instilled in the lungs of mice intratracheally (i.t.). One hour later, cardiolipin was administered i.t. into half of these mice. Inflammation and lung leakage, both markers of pneumonia, were examined by assaying myeloperoxidase (MPO) activity, a measure of neutrophil numbers, and albumin level respectively in the bronchoalveolar lavage (BAL) fluid collected from the mice. While both MPO activity and lung albumin level initially increased in the BAL fluid of mice that received LPS alone as assessed on day 3 after treatment with LPS, both of these parameters were significantly lower on day 6 following LPS treatment (Fig. 2b,c). In the mice that received both LPS and cardiolipin, on day 3 post-treatment, both MPO activity and albumin level in the BAL fluid were higher than that in mice treated with LPS alone and the decrease in these measures was non-significant after day 6 in contrast to that observed in the LPS-treated group (Fig. 2b,c). Mice treated with LPS followed by cardiolipin showed significantly higher levels of the pro-inflammatory cytokines TNF and IL-6 but reduced level of the anti-inflammatory cytokine IL-10 in their lungs as compared with those treated with LPS alone (Fig. 2c).

Lung tissue resident CD11b^+^Ly6G^int^Ly6C^lo^F4/80^+^ cells, resembling MDSCs, produce IL-10 that serves to curtail inflammation during bacterial infection^16^,^23^. As shown in Supplementary Fig. 1, this cell is the major source of the net IL-10 produced in the lungs of mice after LPS treatment. Flow cytometric analysis of lung MDSCs from LPS+cardiolipin-treated mice showed marked suppression of intracellular IL-10 compared with expression in the same cells isolated from mice treated with LPS alone (Fig. 2d). To test whether cardiolipin is actively taken up by lung MDSCs, LPS was delivered i.t. into mice and after 48 h, fluorescently labelled cardiolipin was instilled i.t. into the mice. When examined by confocal microscopy, sorted MDSCs from the lungs of these mice showed uptake of cardiolipin (Supplementary Fig. 2a). Sorted MDSCs from LPS-treated mice also showed suppression of IL-10 when incubated *ex vivo* with both LPS plus cardiolipin (Supplementary Fig. 2b), suggesting a direct suppressive effect of cardiolipin on IL-10 production from MDSCs.

### WT but not IL-10-deficient MDSCs resolve lung inflammation

Since our data suggested that cardiolipin-mediated suppression of IL-10 production from lung MDSCs leads to non-resolving lung inflammation, we wondered whether adoptive transfer of *in vitro* generated MDSCs would minimize lung inflammation and restore tissue homeostasis in mice treated with both LPS and cardiolipin. To this end, bone marrow cells were cultured with GM-CSF and LPS for nine consecutive days. CD11b^+^Ly6G^int^F4/80^+^ cells producing a high level of IL-10 were sorted^14^,^16^ (Supplementary Fig. 3) and adoptively transferred into mice that had received both LPS and cardiolipin the day before. Change in body weight was then monitored in all mice. Although there was an initial loss of body weight in mice that received LPS alone, the mice regained their weight from day 3 onwards. However, the mice treated with both LPS and cardiolipin continued to lose weight until day 6 when they needed to be euthanized (Fig. 2e). Mice that received phosphate-buffered saline (PBS) or cardiolipin alone showed minimal or no change in body weight (Fig. 2e). Mice that received MDSCs generated from bone marrow cells of wild-type (WT) mice showed a significant gain of their initial body weight (Fig. 2e) along with reduced cellular infiltration in the lung on day 6 after LPS+cardiolipin treatment as compared with LPS+cardiolipin-treated mice that did not receive the cells (Fig. 2f). Mice that received the MDSCs also showed decreased MPO activity and protein leak on day 6 following LPS+cardiolipin treatment (Fig. 2g,h). However, MDSCs generated from *Il10*^−/−^ mice failed to mitigate any of the inflammation parameters (Fig. 2e–h). The IL-10 level was higher in the lungs of LPS+cardiolipin-treated mice that received MDSCs generated from WT mice as compared with those that received cells generated from *Il10*^−/−^ mice (Fig. 2i). Thus, overall, the mice that received WT MDSCs displayed improvement in all outcome measures compared with those that received IL-10-deficient MDSCs. These data further strengthened our hypothesis that IL-10 from MDSCs is a critical factor for resolution of lung inflammation following bacterial infection as also shown in our earlier study^16^. However, given that the IL-10-deficient MDSCs had a small inhibitory effect on MPO activity and BAL albumin levels on day 6, it is likely that other mediators produced by these cells also contribute to resolution of inflammation.

### PPARγ mediates LPS-induced IL-10 production in lung MDSCs

**Figure 3:**
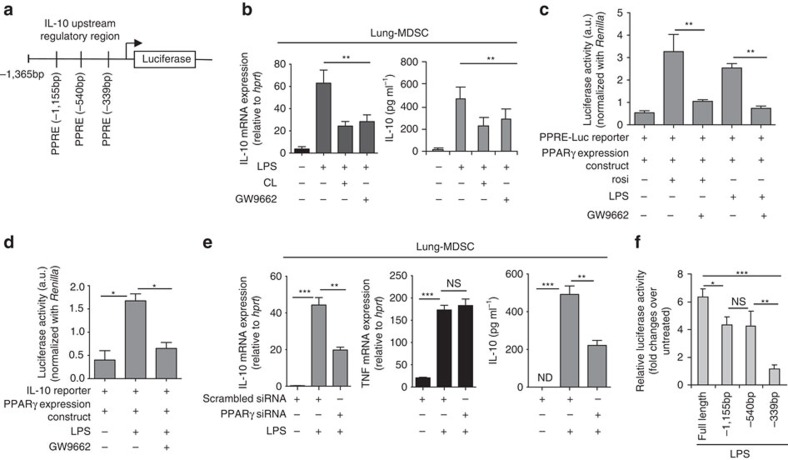
PPARγ is critical for IL-10 regulation in lung MDSCs. (**a**) Schematic of IL-10 upstream regulatory region showing multiple PPAR/RXR response elements (PPAR-RE). (**b**) Lung MDSCs were treated *ex vivo* with LPS (1 μg ml^−1^) either alone or in combination with cardiolpin (10 μg ml^−1^)±GW9662 (10 μM) for 6 h, cardiolipin being added 1 h post LPS treatment. Levels of IL-10 mRNA and secreted IL-10 in the culture supernatant were measured by qPCR (left panel) and ELISA (right panel) respectively. Addition of cardiolipin alone did not promote IL-10 gene expression (data not shown). (**c**) RAW 264.7 cells were transfected with both PPRE-Luciferase reporter construct and PPARγ expression vector. Twenty-four hour post-transfection, cells were stimulated with rosiglitazone (rosi) or LPS± GW9662. Two hour post-stimulation, reporter activity was measured. (**d**) RAW 264.7 cells were transfected with both IL-10 reporter and PPARγ expression constructs. Twenty-four hour post-transfection, cells were stimulated as indicated and reporter activity was measured. (**e**) *Il10* and *Tnf* mRNA expression in lung MDSCs isolated from LPS-treated mice and transfected (1 × 10^6^ cells per condition) with scrambled or *pparg*-targeted siRNA (10 nM) was studied by qPCR (left and middle panel). IL-10 protein in culture supernatant was measured by ELISA (right panel). (**f**) Reporter assay of peritoneal macrophages transfected with plasmid constructs containing WT or deletion mutants of the IL-10 promoter (WT and mutants with deletions of indicated PPAR/RXR sites) linked to the luciferase gene. Statistical significance was calculated using one-way ANOVA with Bonferroni's *post hoc* test for multiple pairwise comparisons. Data shown are mean±s.d. and all data are representative of two independent experiments. *P≤0.05, **P≤0.01 and ***P≤0.001; NS, not significant.

We next explored the mechanism underlying suppression of IL-10 production from lung MDSCs in the presence of cardiolipin. Different transcription factors have been associated with regulation of IL-10 gene expression in various cell types^24^. Although IL-10 was previously shown by us to be an important mediator of lung MDSC function^14^,^16^,^23^, the regulatory elements in the IL-10 promoter that control its expression in the lung MDSCs were not elucidated. Given that the focus of our study was lung inflammation and its resolution, using Ingenuity Knowledge Base (Qiagen) we first identified 718 molecules that have been previously associated with lung inflammation. Since our data suggested that cardiolipin transcriptionally modulates IL-10 expression, we applied the ‘build tool' of Ingenuity Pathway Analysis (IPA) (Qiagen) to reduce the list of 718 molecules to include only transcription factors, ligand-dependent nuclear receptors and cytokines. Since cardiolipin is a lipid molecule, we next used IPA's ‘path explorer' tool to explore the factors involved in lipid-mediated regulation of IL-10 gene expression. This search identified PPARγ as the only transcription factor associated with IL-10 production. Analysis of the upstream regulatory region (URR) in the IL-10 gene also revealed multiple PPARγ response elements (PPRE) (Fig. 3a). Indeed, treatment of lung MDSCs with rosi promoted IL-10 production from the cells (Supplementary Fig. 4a). In published literature, phospholipids have been shown to function as endogenous regulators of PPARγ activity^25^,^26^. As shown in Fig. 3b, use of the specific inhibitor of PPARγ, GW9662, suppressed IL-10 mRNA and protein levels in the lung MDSCs to the same degree as cardiolipin. To be certain that GW9662 specifically inhibits PPARγ activity, we performed a PPRE-luciferase reporter assay in RAW 264.7 cells transfected with both PPRE-luciferase reporter construct and a PPARγ expression vector. Transfected cells were treated with either rosi or LPS alone or in combination with GW9662. We observed a significant reduction in reporter activity with GW9662 in both rosi- and LPS-treated cells suggesting specific inhibition of PPARγ activity with GW9662 (Fig. 3c). In a similar fashion, cardiolipin also inhibited reporter activity induced by a combination of rosi and LPS (Supplementary Fig. 4b). Next we studied the effect of GW9662 on IL-10 promoter activity to investigate whether PPARγ is involved in regulating IL-10 production post-LPS stimulation. To this end, we transfected RAW 264.7 cells with an IL-10 reporter construct along with PPARγ expression vector. We observed a marked reduction in IL-10 reporter activity in the cells treated with both LPS and GW9662 as compared with cells treated with LPS alone, suggesting involvement of PPARγ in LPS-induced IL-10 (Fig. 3d). Also, knocking down PPARγ in lung MDSC cells by PPARγ-specific siRNA (Supplementary Fig. 4c) significantly inhibited LPS-induced IL-10 production, while TNF expression remained unchanged (Fig. 3e). IL-10 reporter assay with WT (extending to −1,365 bp in the URR) and mutant reporter constructs containing deletions of individual PPREs within the context of the full length promoter revealed that the PPRE at the −339 bp location was critical for optimal IL-10 promoter activation (Fig. 3f). Collectively, these data suggested an important role for PPARγ in IL-10 gene expression in lung MDSCs as well as in peritoneal macrophages. These data were in line with findings in previous *in vivo* studies where PPARγ activation by a synthetic ligand^27^ or by a specific gut bacterium^28^ increased IL-10 production although the regulatory mechanism was not explored in these studies.

**Figure 4:**
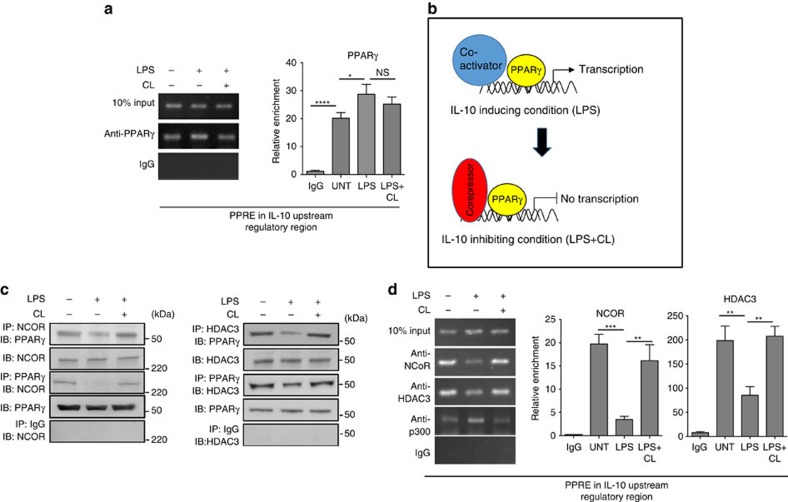
Cardiolipin promotes association of corepressor complex with PPARγ. Lung MDSCs were treated *ex vivo* with LPS (1 μg ml^−1^)±cardiolipin (10 μg ml^−1^) for 2 h and binding of PPARγ to the IL-10 promoter and association with corepressor proteins were studied. (**a**) ChIP assay was performed using PPARγ-specific antibody. Immunoprecipitated chromatin was used as template to amplify the region containing the PPRE at −339 bp in the IL-10 URR (left panel). qPCR quantification of three independent ChIP experiments is shown (right panel). (**b**) Schematic of hypothesis of IL-10 regulation in lung MDSCs by LPS±cardiolipin. (**c**) NCOR- or HDAC3-associated protein complexes were immunoprecipitated using their respective antibodies and probed with PPARγ-specific antibody or *vice versa*. (**d**) ChIP assay using NCOR-, HDAC3- or p300-specific antibody (left panel). qPCR quantitation of three independent ChIP experiments is shown (right panel). Statistical significance was calculated using one-way ANOVA with Bonferroni's *post hoc* test for multiple pairwise comparisons. Data shown are mean±s.d. and all data are representative of two independent experiments. *P≤0.05, **P≤0.01, ***P≤0.001 and ****P≤0.0001; NS, not significant.

To directly demonstrate recruitment of PPARγ to the IL-10 URR in lung MDSCs, we performed a chromatin immunoprecipitation (ChIP) experiment. Although a moderate increase in enrichment of PPARγ was observed in LPS-treated samples, both untreated as well as LPS+cardiolipin-treated samples also showed significant binding of PPARγ to the IL-10 URR (Fig. 4a, left panel). The quantitative analysis of two independent ChIP experiments was performed by qPCR providing similar information (Fig. 4a, right panel). This observation led us to hypothesize that under unstimulated and cardiolipin-treated conditions, PPARγ is recruited to the IL-10 URR in association with corepressors such as NCOR along with HDAC3 (a histone deacetylase). If this were true, while PPARγ would remain bound to the PPREs under these conditions, it would not be functionally active (Fig. 4b). To verify our hypothesis, we first performed immunoprecipitation (IP) experiments using antibodies against NCOR or HDAC3 followed by western blot analysis using anti-PPARγ antibody. Indeed, under unstimulated condition, PPARγ co-precipitated with NCOR and HDAC3 (Fig. 4c). We also performed the reverse experiment using anti-PPARγ for IP followed by western blot analysis using antibodies against NCOR and HDAC3. This association between PPARγ and NCOR/HDAC3 was, however, abolished upon LPS treatment and was restored when cells were treated with LPS+cardiolipin (Fig. 4c). The results of these experiments demonstrated complex formation between PPARγ and NCOR/HDAC3 under conditions where IL-10 expression was low, which were under basal conditions or in the presence of LPS+cardiolipin. To further test our hypothesis, we examined recruitment of NCOR or HDAC3 to the IL-10 URR under these conditions, for which ChIP assay was performed. Recruitment of NCOR and HDAC3 to the IL-10 URR was observed under both basal and LPS+cardiolipin-treated conditions, which was not the case when cells were treated with LPS alone (Fig. 4d). The opposite was true for the recruitment of the coactivator p300 to the IL-10 URR (Fig. 4d). Taken together, these observations showed that cardiolipin prevents LPS-induced dissociation of the corepressor molecule NCOR and of HDAC3 from PPARγ-associated protein complex on the IL-10 URR.

### SUMOylation of PPARγ by cardiolipin blocks IL-10 production

**Figure 5:**
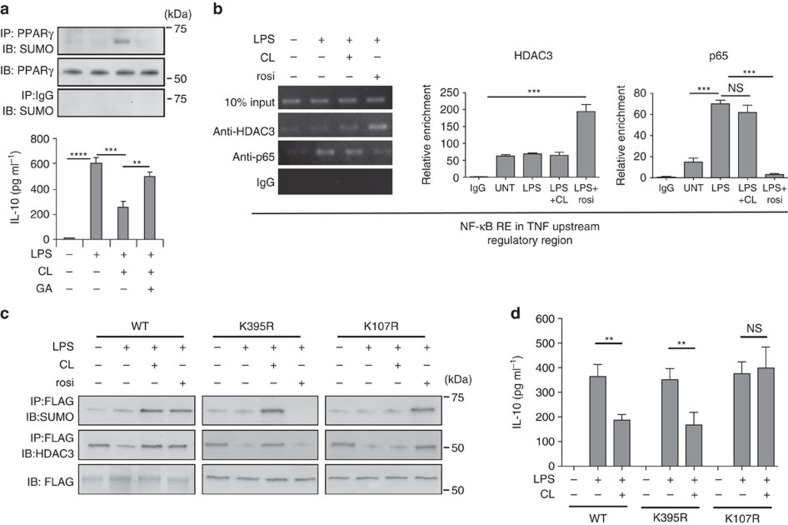
Cardiolipin-induced PPARγ-SUMOylation is required to recruit corepressors to the IL-10 URR. (**a**) Lung MDSCs were treated individually or in combination with LPS, cardiolipin or ginkgolic acid (GA, 50 μM) for 6 h. SUMOylation of immunoprecipitated PPARγ was studied by western blot analysis (upper panel) and IL-10 in the culture supernatant was measured by ELISA (lower panel). (**b**) Lung MDSCs were treated with LPS, cardiolipin or rosiglitazone (rosi) either alone or with the indicated combinations. ChIP assay was performed with antibodies against either HDAC3 or the p65 subunit of NF-κB (RelA). The region containing the NF-κB response element at −214 bp in the TNF promoter was amplified with specific primers. qPCR quantitation represents analysis of two independent ChIP experiments (middle and right panels) (**c**,**d**) RAW macrophages were transfected with plasmid expression constructs containing either FLAG-tagged WT PPARγ gene (left panel) or a variant containing either K395R (middle panel) or K107R (right panel) mutation in PPARγ. Transfected cells were treated individually or in combination with LPS, cardiolipin or rosi. FLAG-tagged PPARγ was immunoprecipitated with anti-FLAG antibody. SUMOylation and HDAC3 association with PPARγ was studied by western blot analysis. (**d**) Secreted IL-10 in the culture supernatant was measured by ELISA. Statistical significance was calculated using one-way ANOVA with Bonferroni's *post hoc* test for multiple pairwise comparisons. Data shown are mean±s.d. and all data are representative of two independent experiments. **P≤0.01 and ***P≤0.001; NS, not significant.

Since SUMOylation of PPARγ has been shown to promote and stabilize corepressor/HDAC-associated PPARγ protein complex by inhibiting proteasome-mediated degradation^21^, we were next interested in determining whether cardiolipin has the ability to induce SUMOylation of PPARγ. A marked increase in SUMOylation of PPARγ was observed in lung MDSCs treated with LPS+cardiolipin, which was largely diminished by the addition of the SUMOylation inhibitor ginkgolic acid (Fig. 5a, upper panel). SUMOylation of PPARγ, however, was not detectable in untreated or LPS alone treated samples. We observed that increased PPARγ SUMOylation was associated with decreased IL-10 expression and inhibition of SUMOylation could overcome the cardiolipin-mediated suppression of IL-10 production (Fig. 5a, lower panel). SUMOylation of PPARγ by its ligand, rosi, has been shown to be critical for suppression of selected NF-κB-regulated genes via transrepression^21^,^29^. Transrepression plays an important role in inhibition of expression of pro-inflammatory genes like TNF and IL-6. However, in our present study, we observed a significant increase in TNF level in whole lung homogenate of mice treated with LPS+cardiolipin with concomitant reduction in IL-10 level (Fig. 2c). These observations in aggregate prompted us to hypothesize that SUMOylation of PPARγ, induced by cardiolipin and rosi occur at distinct amino acid residues in PPARγ and this differential SUMOylation contributes to contrasting physiological outcomes. We then examined the recruitment of HDAC3 to the TNF URR in lung MDSCs treated with either cardiolipin or rosi following NF-κB activation by LPS. Data from ChIP analysis showed marked recruitment of HDAC3 on the NF-κB response element at −214 bp in the TNF URR in the presence of rosi but not cardiolipin (Fig. 5b, left panel). This observation was supported by another ChIP assay where LPS-induced p65 recruitment on the same NF-κB RE of TNF-URR was significantly reduced by rosi treatment but not with cardiolipin (Fig. 5b, left panel). The quantitative analysis of both HDAC3 and p65 ChIP assays performed by qPCR revealed similar trend (Fig. 5b, right panels). Collectively, these data confirmed that cardiolipin and rosi differentially regulate TNF, both involving PPARγ.

Our next task was to investigate whether differential HDAC3 recruitment by cardiolipin versus rosi is due to distinct SUMOylation of PPARγ. The present literature revealed two SUMOylation sites in PPARγ-one at lysine 107 (K107) and the other at lysine 395(K395)^21^,^30^,^31^,^32^,^33^. K395 SUMOylation in PPARγ is involved in transrepression^21^, while SUMOylation at K107 represses gene transcription directly in *cis* by binding to PPREs^33^,^34^,^35^. To investigate which site was utilized by cardiolipin for HDAC3 recruitment to the IL-10 URR, PPARγ expression constructs containing point mutation in the individual SUMOylation sites were introduced in the RAW264.7 macrophage cell line (which lacks endogenous PPARγ expression) and the cells were stimulated with LPS±cardiolipin or rosi. Immunoprecipitated PPARγ was analysed for SUMOylation and HDAC3 association. Cardiolipin treatment following LPS stimulation failed to induce PPARγ SUMOylation and HDAC3 association when the K107 site was mutated (Fig. 5c). However, SUMOylation and HDAC3 recruitment were unchanged when the K395 SUMOylation site was mutated. In contrast, SUMOylation and HDAC3 recruitment were evident with rosi when the K107 site was mutated, but was lost when the K395 position was altered to arginine (Fig. 5c). Figure 5d shows no effect of the K395R mutation on cardiolipin-mediated inhibition of IL-10 production from the macrophages although the inhibition was lost when the K107R mutant was used. The results showed that SUMOylation of PPARγ induced by cardiolipin and rosi involves different amino acid residues in PPARγ and provide a fundamental mechanism for differential TNF regulation by these two agents.

### Cardiolipin metabolite cPA induces SUMOylation of PPARγ

**Figure 6:**
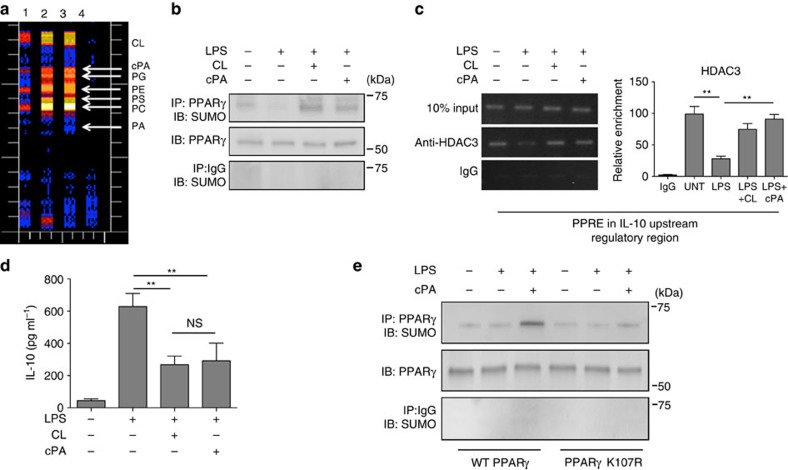
The cardiolipin metabolite cPA induces SUMOylation of PPARγ and recruitment of corepressor complex. (**a**) Lung MDSCs were incubated with ^3^H-labelled cardiolipin for 10, 30 and 60 min and cellular lipids were extracted using the Bligh and Dyer method. Lipids were then resolved by thin layer chromatography to detect individual phospholipids. The ^3^H metabolites of cardiolipin were quantified using a plate reader. Lanes 1, 2 and 3 depict incubation of ^3^H-labelled cardiolipin for 10, 30 and 60 min, respectively, lane 4—blank. (**b**–**d**) Lung MDSCs were treated with either LPS (1 μg ml^−1^) alone or in combination with cardiolipin (10 μg ml^−1^) or cPA (50 μM) and analysed. (**b**) PPARγ was immunoprecipitated and SUMOylation was studied by western blot methods. (**c**) HDAC3 recruitment to the IL-10 URR was investigated by ChIP assay (left panel). qPCR quantitation represents analysis of three independent ChIP experiments (right panel). (**d**) IL-10 in cell-free culture supernatant was measured by ELISA. (**e**) RAW 264.7 cells were transfected with either wild type or mutated (K107R) PPARγ (FLAG-tagged) expression construct. Twenty-four hour post-transfection, cells were stimulated with LPS±cPA as indicated. Two hours post-stimulation, PPARγ was immunoprecipitated and SUMOylation was examined by western blot methods. Statistical significance was calculated using one-way ANOVA with Bonferroni's *post hoc* test for multiple pairwise comparisons. Data shown in all experiments are mean±s.d. and data are representative of two independent experiments. **P≤0.01; NS, not significant.

To understand how extracellular cardiolipin works on PPARγ we analysed the intracellular metabolites of cardiolipin in lung MDSCs. Isolated lung MDSCs were treated with ^3^H-labelled cardiolipin for different lengths of time and intracellular metabolites were analysed by thin layer chromatography. As shown in Fig. 6a, exposure of the cells to extracellular cardiolipin led to an increase in the levels of different lipid moieties including cPA. In view of the fact that cPA is a potent antagonist of PPARγ (ref. 36) we investigated whether like cardiolipin, cPA has the ability to induce SUMOylation of PPARγ with concomitant recruitment of HDAC3 to the IL-10 URR. Towards this end, we exposed lung MDSCs to purified cPA and examined SUMOylation of PPARγ by immunoprecipitation. A marked increase in SUMOylation of PPARγ was noticed in the cells treated with cPA which mimicked that induced by cardiolipin (Fig. 6b). Next we investigated whether cPA-mediated SUMOylation of PPARγ also led to the recruitment of HDAC3 to the IL-10 URR. ChIP analysis of lung MDSCs treated with cPA revealed a similar level of recruitment of HDAC3 to the IL-10 URR as induced by cardiolipin (Fig. 6c, left panel). Quantitation of ChIP experiments performed by qPCR using samples from two independent experiments is shown (Fig. 6c, right panel). Next we investigated the effect of cPA-induced recruitment of corepressors HDAC3 and NCOR on IL-10 expression, in lung MDSC cells. Similar to cardiolipin treatment, a marked suppression in the level of IL-10 was observed in the culture supernatant of cells treated with LPS and cPA (Fig. 6d). Our next goal was to investigate whether both cPA and cardiolipin use the same K107 amino acid residue of PPARγ for SUMOylation. With this objective, we transfected RAW264.7 macrophage cell line with either wild type or mutated PPARγ expression vector in which the K107 residue was mutated to arginine (K107R). Cells were stimulated with or without LPS±cPA, PPARγ was immunoprecipitated and SUMOylation was studied by western blotting methods. As shown in Fig. 6e, the marked induction of PPARγ SUMOylation by cPA treatment was reduced when cells were transfected with the mutant (K107R) PPARγ expression vector demonstrating that both cardiolipin and cPA use the same amino acid residue for PPARγ SUMOylation (Figs 5c and 6e). As shown in Supplementary Fig. 5, the lung MDSCs express the enzymes autotaxin and PLD2 that may be involved in the metabolism of cardiolipin to cPA. Collectively, these data suggest the involvement of cPA as a cardiolipin metabolite that mediates its biological effect of inhibition of IL-10 expression in MDSCs.

### HDAC inhibition restores IL-10 with resolution of pneumonia

**Figure 7:**
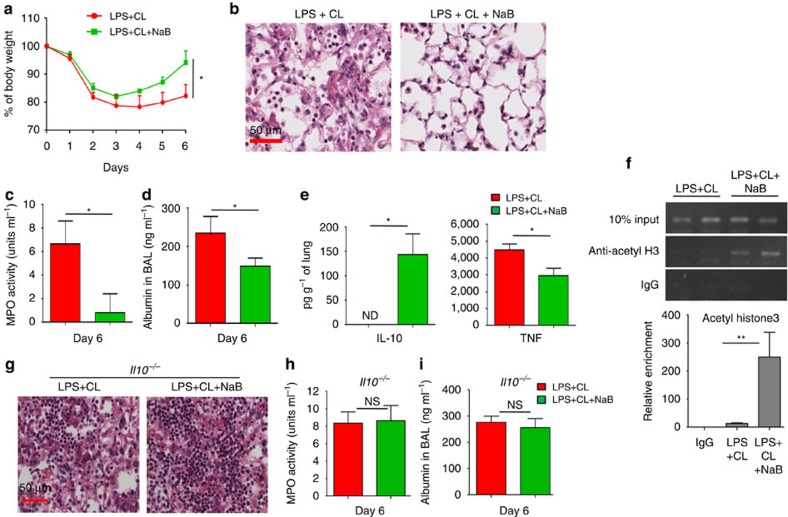
Inhibition of HDAC3 by a short-chain fatty acid promotes resolution of lung inflammation. C57BL/6J mice were treated i.t with both LPS (20 μg per mouse) and cardiolipin (100 μg per mouse). Day 1 post-treatment, mice were randomly divided into two groups. One group was treated i.p. with an HDAC3 inhibitor, sodium butyrate (NaB) (200 mg kg^−1^ body weight) for five consecutive days while the other group received only vehicle (PBS). Day 6 post-treatment, the following parameters of inflammation were studied: (**a**) Change in body weight, (**b**) lung pathology by H&E staining of lung tissue sections, (**c**) MPO activity in BAL fluid, (**d**) BAL albumin level and (**e**) Secreted cytokines (IL-10 and TNF) assayed in lung homogenates using magnetic bead-based assay. (**f**) Lung MDSCs were isolated from the two groups of mice and ChIP assay was performed to study recruitment of acetyl-histone to the IL-10 URR (upper panel). qPCR quantitation represents analysis of two independent ChIP experiments (lower panel). (**g**–**i**) *Il10*^−/−^ mice were treated i.t. with both LPS and cardiolipin as described above. Day 1 post-treatment, mice were randomly divided into two groups. One group was treated (i.p.) with an HDAC3 inhibitor, sodium butyrate (NaB), while the other group received only vehicle (PBS). Day 6 post-treatment, the following parameters of inflammation were studied: (**g**) lung pathology by H&E staining of lung tissue sections, (**h**) MPO activity in BAL fluid, (**i**) BAL albumin level by ELISA. Statistical significance was calculated using Student's unpaired two-tailed *t* test for comparisons involving two groups or one-way ANOVA with Bonferroni's *post hoc* test for multiple pairwise comparisons. The experiment was initiated with 8 mice in LPS+cardiolipin group and 6 mice in LPS+cardiolipin+NaB group with 3 and 4 mice respectively in each group available for analysis at the conclusion of the experiment. Data shown are mean±s.d. and are representative of two independent experiments. *P≤0.05, **P≤0.01; NS, not significant.

Because our data suggested that the recruitment of HDAC3 to the IL-10 URR is critical for cardiolipin-mediated IL-10 suppression, we investigated whether inhibition of HDAC activity *in vivo* using the short-chain fatty acid, sodium butyrate (NaB)^37^, could overcome IL-10 suppression. To this end, we treated mice with NaB for five consecutive days, initiated a day after treatment with LPS+cardiolipin. Among the LPS+cardiolipin-treated mice, the group of mice that received NaB showed significant gain in body weight (Fig. 7a), restoration of normal lung histology (Fig. 7b), reduced MPO activity in BAL fluid (Fig. 7c), significant reduction in lung leakage (Fig. 7d) and increased IL-10 but decreased TNF levels in the lung (Fig. 7e) as compared with the vehicle-treated group. ChIP analysis of lung MDSCs isolated from NaB-treated mice showed significantly greater recruitment of acetylated Histone 3 (acetyl H3) to the IL-10 URR (Fig. 7f, upper panel). Quantitation of ChIP experiments performed by qPCR yielded similar data (Fig. 7f, lower panel). This observation was in line with the increased level of IL-10 in the whole lung (Fig. 7e). Since NaB inhibits multiple HDACs including HDAC3, we were interested to determine the extent to which the therapeutic potential of NaB was dependent on its role in rescuing IL-10 suppression. To this end we treated *Il10*^−/−^ mice with NaB for five consecutive days, initiated a day after treatment with LPS+cardiolipin. In NaB-treated *Il10*^−/−^ mice, we did not observe improvement of lung histology as compared with vehicle-treated *Il10*^−/−^ mice (Fig. 7g). Moreover, both groups of mice showed comparable BAL MPO activity (Fig. 7h) and lung leakage (Fig. 7i). These data suggest that although NaB inhibits multiple HDACs including HDAC3, in our experimental model the therapeutic potential of NaB is primarily due to its role in rescuing IL-10 suppression by inhibiting HDAC3.

**Figure 8:**
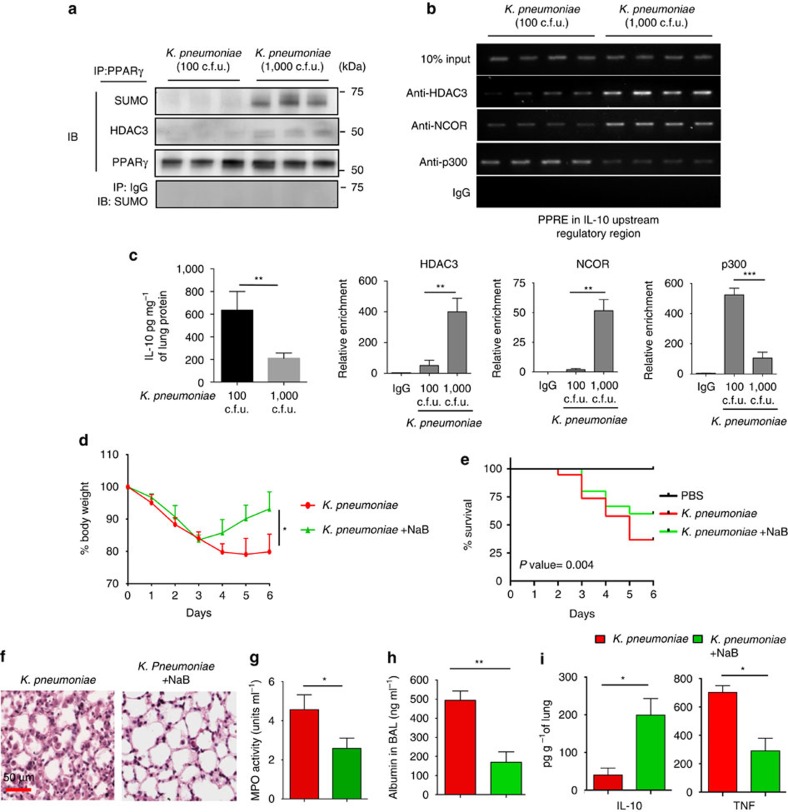
Infection with high dose of *K. pneumoniae* recruits corepressor complex to the IL-10 URR and amelioration of pathogenicity by short-chain fatty acid. (**a**–**c**) C57BL/6J mice were infected i.t. with either low dose (100 c.f.u.) or high dose (1,000 c.f.u.) of *K. pneumoniae* (*n*=3–4 mice per group at the conclusion of experiment). Four days post-infection, lung MDSCs were purified. (**a**) Coimmunoprecipitation and (**b**) ChIP experiments were performed using NCOR-, HDAC3- or p300-specific antibodies. Data shown are representative of two independent experiments. qPCR quantitation represents analysis of two independent ChIP experiments (lower panel). (**c**) IL-10 protein level in the lung on day 6 was measured by magnetic bead-based cytokine assay. Data shown are mean±s.d. and representative of two independent experiments. (**d**–**i**) C57BL/6J mice were infected with 1,000 c.f.u. of *K. pneumonia*. Day 1 post-infection, mice were randomly divided into two groups. One group was treated i.p. with NaB (200 mg kg^−1^ body weight) for five consecutive days while the other group received only vehicle (PBS). Day 6 post-treatment, the following parameters of inflammation were studied: (**d**) Change in body weight. (**e**) Percentage of survival represented by Kaplan–Meier survival curves of PBS-treated mice or mice infected with Kleb±NaB treatment. Data shown are mean±s.d. from three pooled experiments with *n*=12 mice in PBS group, 15 in bacteria only group and 20 in bacteria+NaB group. The *P* value was calculated using log-rank (Mantel-Cox) test. (**f**) Lung pathology by H&E staining of lung tissue sections. (**g**) MPO activity in BAL fluid, (**h**) BAL albumin level by ELISA, and (**i**) secreted cytokines assayed in lung homogenates using magnetic bead-based assay. Experiments in **c**,**d** and **f**–**i** were initiated with *n*=8 and 6 mice in *Klebsiella* and *Klebsiella*+NaB groups, respectively. At the conclusion of the experiment, 3 mice in bacteria only group and 4 mice in bacteria+NaB group were available for analysis. Statistical significance was calculated using Student's unpaired two-tailed *t* test for comparisons involving two groups or one-way ANOVA with Bonferroni's *post hoc* test for multiple pairwise comparisons. Data shown are mean±s.d. and are representative of two independent experiments. *P≤0.05, **P≤0.01, ***P≤0.001.

With our ultimate goal being to understand the mechanism of compromised IL-10 expression under conditions of non-resolving bacterial pneumonia, we verified involvement of this mechanism in mice infected with *K. pneumoniae*. IP performed with lung MDSCs isolated from mice infected with high dose (1,000 c.f.u.) of *Klebsiella* revealed increased SUMOylation of PPARγ which was not evident in mice treated with a low dose (100 c.f.u.) of bacteria (Fig. 8a). Increased SUMOylation was coupled with augmented association of PPARγ with HDAC3 in the same group of mice (Fig. 8a). Also, when mice were infected with the low dose of bacteria (100 c.f.u.), HDAC3 and NCOR recruitment to the IL-10 URR was lower compared with recruitment of the coactivator, p300 (Fig. 8b). However, infection with a higher dose of bacteria (1,000 c.f.u.) yielded opposite data with increased recruitment of NCOR/HDAC3 and decreased recruitment of p300 (Fig. 8b). Quantitation of ChIP assays for HDAC3, NCOR and p300 was performed by qPCR using samples from two independent experiments and the results are shown below Fig. 8b. Similarly, high but not low dose of bacteria suppressed IL-10 levels in the lung (Fig. 8c). We next examined the effect of NaB in mice infected with *K. pneumonia* (1,000 c.f.u.). Similar to data obtained in mice treated with LPS+cardiolipin, NaB significantly inhibited loss in body weight, caused increased survival, inhibited lung pathology and significantly reduced lung leakage, the combination was the most effective (Fig. 8d–g). NaB treatment also led to an increase in lung IL-10 level and a decrease in TNF level (Fig. 8h). Taken together, these data reveal a dynamic interplay between PPARγ SUMOylation and corepressor/HDAC and coactivator to regulate IL-10 production, which is compromised by cardiolipin and the ability of NaB to overcome the cardiolipin-induced adverse effects.

## Discussion

Non-resolving lung inflammation induced by bacterial infection is still a leading cause of morbidity and mortality worldwide. Continuous stimulation through TLRs, increased cell death and poor clearance of apoptotic neutrophils are considered to be the major contributors to aggravated lung inflammation^1^. However, it is not well understood why anti-inflammatory responses fail under conditions of non-resolving pneumonia. Here we show that the phospholipid cardiolipin, previously shown to be elevated in the lungs of humans with bacterial pneumonia^17^, and also in the lungs of mice infected by bacteria (present study), blunts IL-10 production from lung MDSCs and perpetuates lung inflammation. We expect that non-resolving lung inflammation associated with other Gram-negative bacteria would involve similar mechanisms of corepressor recruitment to the IL-10 promoter since our observations were essentially similar with LPS+cardiolipin and *K. pneumoniae*. The immunomodulatory role of MDSCs in cancer is well recognized where they suppress anti-tumour immune responses and promote tumour progression and metastasis^38^,^39^. However, a beneficial role of MDSCs in terminating infection-associated inflammation is less studied but being increasingly appreciated^23^. We show an important role of PPARγ in promoting IL-10 gene expression in the MDSCs and the ability of cardiolipin to impair this process by inducing SUMOylation of PPARγ causing recruitment of an NCOR/HDAC corepressor complex to the IL-10 promoter without affecting the TNF promoter. The PPARγ SUMOylation at K107 identified in our study is distinct from the well-described K395 SUMOylation that can be induced by PPARγ agonists such as the thiazolidinedione, rosi. Our data show that cardiolipin differentially influences expression of anti- (IL-10) versus pro- (TNF)-inflammatory cytokines via selective recruitment of corepressor complex to the IL-10 promoter region. We show beneficial effect of HDAC inhibition using a short-chain fatty acid, NaB, in the lungs of infected mice, which restored IL-10 levels but suppressed TNF levels with accompanying gain in body weight and survival of the animals.

In our efforts to determine how a high level of cardiolipin generated in the lungs of mice infected by bacteria inhibits IL-10 production, we detected accumulation of intracellular metabolites like cPA when cells were exposed to cardiolipin, which highlights a mechanism by which extracellular cardiolipin can exert deleterious effects on important immunoregulatory mechanisms. This is because cPA was previously shown to inhibit PPARγ activation by rosi due to competitive binding of cPA to the active site of PPARγ (ref. 36). We show similar inhibitory effects of cardiolipin and cPA on IL-10 production from lung MDSCs, both being lost when K107 in PPARγ is mutated, this residue being a known SUMOylation target and is involved in *cis*-activating functions of PPARγ (refs 33, 34, 35). Although not the goal of the present study, since cardiolipin can be metabolized to cPA, we do expect cardiolipin to function as an antagonist of rosi-activated PPARγ as previously reported^36^. It is interesting to note similar end results of targeting cPA in the two studies; while blocking cPA generation using PLD2 shRNA relieved cPA-mediated suppression of the PPARγ target genes *fabp4* and *cd36* in the previous study^36^, in our experiments, blocking cardiolipin/cPA function by blocking K107 SUMOylation using ginkgolic acid relieved suppression of IL-10 production (Fig. 5a).

The key finding in our study is the ability of cardiolipin to inhibit anti-inflammatory mechanisms such as IL-10 production from lung MDSCs, which is the principal source of IL-10 during bacterial infection. Compromised IL-10 production observed in the presence of cardiolipin was associated with higher numbers of infiltrating cells, which persisted in the lung. Thus, cardiolipin can impair resolution of inflammation by antagonizing a key anti-inflammatory immune response during bacterial infection^16^. Cardiolipin has been shown to directly exert pro-inflammatory effects by promoting NLRP3 inflammasome activation^40^ and in this context too IL-10 serves an important anti-inflammatory function by inhibiting NLRP3-mediated inflammasome activation^41^,^42^.

Although PPARγ activation by a synthetic ligand^27^ or a gut bacterium^28^ was previously associated with increased IL-10 production in the gut mucosa, our study provides direct molecular proof of PPARγ utilization during IL-10 gene expression. Since IL-10 plays a central role in resolution of inflammation, its regulation has been studied in several immune cell types including T cells, dendritic cells and macrophages^43^. Multiple transcription factors including SP1 (ref. 44), MAF^43^,^45^,^46^, GATA-3 (refs 47, 48), ATF2, CREB^49^ and NF-κB^50^,^51^ have been implicated in IL-10 gene expression, which collectively illustrates cell-specific mechanisms of IL-10 gene transcription. IL-10 regulation in lung MDSCs, however, is not well understood. In the present study, we have identified a novel mechanism of IL-10 gene expression involving PPARγ that does not involve the well-studied transrepression function of PPARγ and yet contributes to the anti-inflammatory actions of PPARγ by promoting IL-10 production. Of note, while our study demonstrates an essential role of PPARγ in mediating IL-10 gene expression in the lung MDSCs, it is likely that PPARγ acts in concert with other transcription factors to induce optimal transcription of IL-10. We have observed that under basal conditions, PPARγ is constitutively bound to the IL-10 upstream regulatory region along with the corepressors HDAC3 and NCOR. Interaction of members of the nuclear receptor family with corepressor molecules under basal conditions has been reported for the thyroid hormone receptor and the retinoid X receptor^52^,^53^,^54^,^55^ but has not been demonstrated for PPARγ heretofore. This mechanism plays a critical role in restricting basal IL-10 expression, which has physiological significance. Our study and those of others have shown that during bacterial infection, early expression of IL-10 is detrimental since it inhibits the recruitment of leukocytes, which is necessary for bacterial clearance^8^,^16^,^56^. However, delayed expression of IL-10 is equally important to terminate the ongoing inflammatory response to prevent tissue injury^16^.

Anti-inflammatory function of PPARγ has been studied in multiple inflammatory diseases including rheumatoid arthritis, diabetes, inflammatory bowel disease and atherosclerosis in which PPARγ agonists such as rosi have been used^57^,^58^,^59^. In these studies, transrepression of NF-κB-regulated genes by PPARγ was shown to be the key anti-inflammatory mechanism. As previously shown, rosi-mediated SUMOylation at K395 of PPARγ is important for transrepression functions of PPARγ that involves recruitment of corepressor-associated PPARγ to NF-κB binding sites through protein–protein interactions thereby blocking transactivation functions of NF-κB (refs 21, 29). Major pro-inflammatory genes that are regulated by transrepression include TNF, iNOS and IL-6. However, in our present study we did not observe any suppression of TNF and IL-6 with cardiolipin treatment- instead, the levels of both cytokines significantly increased in cardiolipin+LPS-treated mice as compared with that in mice treated with LPS alone. These results showed that SUMOylation of PPARγ induced by cardiolipin and that induced by rosi are distinct. Here, it is important to note that basal regulation of IL-10 does not require PPARγ SUMOylation while it is absolutely necessary to restrict its expression in the presence of cardiolipin. Cardiolipin promotes enrichment of corepressor/HDAC complex over that of a co-activator on the IL-10 promoter but spares the TNF promoter, the net effect being persistent inflammation. Emerging literature suggests an important role of post-translational modifications of PPARγ in regulating PPARγ activity impacting multiple disease conditions^31^,^57^,^60^. However, a role for PPARγ post-translational modification in promoting non-resolving lung inflammation by altering the coactivator:corepressor balance at the regulatory region of an important immunosuppressive gene has not been previously described.

Health benefits of short-chain fatty acids (SCFAs) are being increasingly appreciated where the major focus is improvement of gut-health^61^,^62^. Here we show that use of the SCFA NaB is highly effective in the context of persistent lung inflammation during bacterial pneumonia. A protective effect of NaB was also observed in severe burn-induced lung injury in rats^63^. Our study highlights a new opportunity for future drug development using SCFAs as an adjunct therapy to ameliorate non-resolving pneumonia by maintaining IL-10 expression and reducing expression of pro-inflammatory cytokines like TNF.

## Methods

### Mice

To address our objective of how cardiolipin impairs resolution of lung inflammation we set up experimental mouse groups based on our original pilot experiments of bacterial infection using an LD50 dose in which assuming a two-sided *α* of 0.05, four animals in each group are required to detect a 15–20% reduction in body weight on day 6 after infection with 80% power. We also used a model of non-resolving lung inflammation using a low dose of LPS in combination with cardiolipin. Having used a bacterial LD50 dose for infection after day 6 with similar inflammation induced by LPS+cardiolipin, we always initiated our experiments with eight animals in each group to accommodate attrition during the course of the study. We also conducted experiments to study the effect of the HDAC inhibitor, NaB, on lung inflammation and loss in body weight of the animals after infection or treatment with LPS+cardiolipin. Mice were randomized for treatment. However, the experimenter was not blinded to the identity of the groups. Since we expected 50% loss of animals due to use of LD50 dose of infection, animals that fit criteria for euthanasia after day 3 of infection, were killed in compliance with a protocol approved by our Institutional Animal Care and Use Committee at the University of Pittsburgh.

Male C57BL/6 J mice, and *Il10*^−/−^ mice on C57BL/6 J background (Stock # 002251), were purchased from The Jackson Laboratory (Bar Harbor, ME). All mice were housed under pathogen-free conditions and were used between 6 and 10 weeks of age. All studies with mice were approved by the Institutional Animal Care and Use Committee at the University of Pittsburgh.

### Reagents

Rosiglitazone (Cat # R2408), GW9662 (Cat # M6191), ginkgolic acid (Cat # 75741), sodium butyrate (Cat # B5887) and LPS (from *Escherichia coli,* strain O26:B6) (Cat # L8274), were obtained from Sigma. Cardiolipin was purchased from Avanti polar lipids, (Cat # 840012C), the major cardiolipin species being (18:2)_4_ or tetralinoleyl cardiolipin. GM-CSF was purchased from Peprotech (Cat # 300-03). Antibodies against HDAC3 (polyclonal, Cat # ab7030), NCOR (polyclonal, Cat # ab24552), NF-κB (p65) (polyclonal, Cat # ab7970), p300 (clone NM11, Cat # ab3164), acetyl-Histone3 (polyclonal, Cat # ab4441) and PPARγ (polyclonal, Cat # ab19481) were purchased from Abcam and the anti-SUMO antibody (polyclonal, Cat # 4930) was from Cell Signaling technology, USA. Antibodies were used at 1:1,000 dilution for western blotting and at 1:50 dilution for immunoprecipitation and ChIP assays. For flow cytometry, antibodies used were against IL-10 (clone JESS-16E3, either PE-conjugated, Cat # 561060, from BD Biosciences used at 1:200 dilution or PercP-Cy5.5-conjugated, Cat # 505027 from Biolegends used at 1:200 dilution), CD11b (PerCP-Cy5.5-conjugated, clone M1/70, Cat # 561114, used at 1:500 dilution), Ly6C (FITC-conjugated, clone AL-21, Cat # 553104, at 1:500 dilution), and Ly6G (APC-conjugated, clone 1A8, Cat # 560599) all purchased from BD Biosciences, and F4/80, (clone BM8, either AF488-conjugated clone BM8, Cat # MF48020 from Biolegend or PE-conjugated, Cat # MF48004 from Life Technologies both used at 1:400 dilution).

### Cell isolation and sorting

To isolate lung MDSCs, mice were treated with LPS (10 μg per mouse) for 3 consecutive days. Twenty-four hours post-treatment, the lungs of anaesthetized mice were perfused with sterile PBS, removed and digested using methods previously described^16^. Briefly, lungs were dissociated in a collagenase-DNase suspension on a gentle MACS Dissociator (MiltenyiBiotec) according to the manufacturer's protocol. Single-cell suspensions were obtained by passing the dissociated tissue through a 70 μm cell strainer (BD Falcon) and washed with PBS containing 2% FBS. CD11b^+^ cells were enriched using CD11b antibody-coated magnetic beads from Miltenyi Biotec. Enriched cells were sorted to 90–95% purity by flow cytometry using fluorochrome-labelled CD11b, F480 and Ly6G antibodies (BD Biosciences). Thioglycollate-elicited peritoneal macrophages were isolated day four post-treatment from mice injected intraperitoneally (i.p.) with 2% thioglycollate (Sigma, Cat #T9032).

### *In vivo* and *ex vivo* treatments

Male C57BL/6J mice were treated i.t. with either LPS (20 μg per mouse), alone or in combination with cardiolipin (100 μg per mouse). Cardiolipin was administered 1 h post LPS instillation. Our experiments having shown cardiolipin levels in the lungs of infected mice in the range of 10–50 μg per lung, we used a dose of 100 μg of cardiolipin to maximize availability of cardiolipin in the lungs of all mice after i.t. instillation. Lung MDSCs were treated *ex vivo* with or without LPS (1 μg ml^−1^). One hour post LPS-treatment, cardiolipin (10 μg ml^−1^) was added to the culture medium. For ChIP, immunoprecipitation and western blot experiments, cells were harvested 2 h post cardiolipin treatment while for ELISA, culture supernatants were collected 6 h post cardiolipin stimulation.

### LC/MS analysis of cardiolipin

Mice were either left uninfected or infected with 1,000 c.f.u. of *K. pneumoniae*. Day 3 post infection lipids were extracted from lung homogenates using the Folch protocol^64^ and phospholipid phosphorus was estimated by a micro-method^65^. LC/MS of cardiolipins was performed as previously described^66^. Briefly, LC/MS in negative mode was performed using a DionexUltimate 3,000 HPLC coupled on-line to a Q-Exactive hybrid quadrupole-orbitrap mass spectrometer (ThermoFisher Scientific, San Jose, CA, USA). Total lipids were separated on a normal phase column (Silica Luna 3 μm, 100 Å, 150 × 2 mm, (Phenomenex, Torrance, CA, USA) with flow rate of 0.2 ml min^−1^ using gradient solvents containing 5 mM CH_3_COONH_4_ (A–n-hexane:2-propanol:water, 43:57:1 (v/v/v) and B—n-hexane:2-propanol:water, 43:57:10 (v/v/v)). Tetra-myristoyl–cardiolipin (Avanti Polar Lipids) was used as an internal MS standard. Oxygenated cardiolipin was analysed by LC/MS as described^66^. Briefly, to prevent lipid oxidation, chromatography was performed under N_2_ on diethylenetriaminepentaacetic acid (DTPA)-treated silica plates (5 × 5 cm, Whatman). LC/MS in negative mode was performed using a Dionex UltimateTM 3,000 RSLCnano system coupled online Q-Exactive hybrid quadrupole-orbitrap mass spectrometer (ThermoFisher Scientific, San Jose, CA, USA) using a C8 column (Luna 3 μm, 100 Å, 150 × 2 mm, Phenomenex, Torrance, CA, USA) with flow rate 0.15 ml min^−1^ using an isocratic solvent system consisting of 2-propanol:water:triethylamine:acetic acid, 45:5:0.25:0.25 (v/v). The resolution was set up at 140,000, which corresponds to 5 p.p.m. in *m*/*z* measurement error. Tetra-myristoyl–cardiolipin (Avanti polar lipids, Alabaster, AL, USA) was used as an internal MS standard. In addition, Tetra-linoleyl–cardiolipin was also utilized as a reference standard to build calibration curves employed for quantitation of all cardiolipin species.

### RNA isolation and qRT-PCR

Lungs were dissociated in TRIzol (Life Technologies) using a high-speed homogenizer. RNA was isolated using RNeasy kit (Qiagen) and treated with RNase-free DNase (Qiagen). cDNA was synthesized using High Capacity cDNA Reverse Transcription kit (Life technologies) according to the manufacturer's instructions. Real-time quantitative PCR was performed using validated TaqMan Gene Expression primer and probe sets (Life Technologies) according to the manufacturer's instructions. Results were analysed using the SDS 2.2.2 software. mRNA expression was calculated using the 2^−ΔCt^ method using *hprt1* as internal reference control.

### Transfection and reporter assay

Cells were transfected with either IL-10-luciferase reporter construct (Adgene) or PPRE-luciferase reporter construct (Qiagen, USA, Cat # CCS-3026L), using Amaxa Mouse Macrophage Nucleofector Kit from Lonza (Cat # VPA-1009) following manufacturer's instructions. Reporter assay was carried out 24 h post-transfection using dual luciferase assay kit (Promega, Cat # E1910) following manufacturer's protocol. Briefly, cells were washed with PBS followed by lysis with 1X passive lysis buffer. The lysate was cleared by brief centrifugation and firefly luciferase reporter activity in the clear supernatant was measured using a luminometer (Veritas, Promega). *Renilla* luciferase activity in the same lysate was used to assess transfection efficiency.

### Immunoprecipitation

Immunoprecipitation assay was performed using Pierce Crosslink Immunoprecipitation Kit (Cat # 26147) following supplier's instructions. Briefly, desired antibodies (10 μg per IP) were adsorbed onto protein A/G agarose and crosslinked. Cells were washed with cold PBS and lysed. Cell lysates were cleared by centrifugation and supernatants were collected. Pre-clearing of cell lysates was done by mixing with washed agarose slurry and incubating for 1 h on a rocker at 4 °C. Protein A/G agarose beads were removed by centrifugation and supernatants were transferred to fresh tubes. Protein concentration was determined by Bradford assay. Six hundred microgram of total protein was incubated with 10 μg of antibody-crosslinked protein A/G agarose overnight on a rocker at 4 °C. After multiple washes, the antigen was eluted with 50 μl of elution buffer and analysed on SDS–PAGE (polyacrylamide gel electrophoresis) gel.

### ChIP assay

ChIP assays were performed using Pierce AgaroseChIP kit (Thermo scientific, Cat # 26156) following manufacturer's instructions. Briefly, thioglycollate-elicited peritoneal macrophages treated with either LPS alone or in combination with cardiolipin were crosslinked using formaldehyde and harvested by scraping. The cell pellet was resuspended in lysis buffer provided with the kit, and chromatin was immunoprecipitated using antibodies against PPARγ, NCOR, HDAC3, p300 or NF-κB (p65). Immunoprecipitated genomic DNA and specific primers (FP 5′ TATCGGACGTTCAACCCAGG 3′; RP 5′ GGCCCTCATCTGTGGATTCC 3′) were used to amplify the region containing the PPRE at −349 bp of the IL-10 promoter. Different primers (FP 5′ CTTCAGCCACTTCCTCCAAG 3′; RP 5′ CATCCATGGGGGAGAACTTA 3′) were used to amplify the region containing the NF-κB response element at −513 bp in the TNF promoter.

### Western blot analysis

Cell lysates were prepared by harvesting cells in cell lysis buffer (Cell Signaling Technology, Cat # 9830S) supplemented with protease inhibitors (Thermo scientific, Cat # 87786). After clearing the lysate by centrifugation, supernatants were collected, and total protein content was measured by Bradford assay. Thirty microgram of total protein was resolved on a 10% SDS–PAGE gel and the proteins were transferred to a polyvinylidenedifluoride membrane. The blot was probed with the specific primary antibody followed by HRP-conjugated secondary antibody (Pierce) and developed using SuperSignal West Femtochemiluminescence substrate (Pierce, Cat # 34095).

### siRNA-mediated knockdown of gene expression

siRNAs specific to PPARγ were purchased from Dharmacon, USA (cat # L-040712-00-0005) while the scrambled siRNAmix was from Santa Cruz Biotechnology, USA. Mice were treated with LPS once over three consecutive days (10 μg each day) and lung MDSCs were isolated 1 day after last treatment and used for transfection (10^6^cells/transfection). Cells were transfected with either specific siRNA or scrambled siRNA using Amaxa Mouse Macrophage Nucleofector Kit from Lonza following manufacturer's instructions. Post-transfection, cells were maintained at 37 °C in the presence of 5% CO_2_. The efficiency of gene knockdown was verified by qRT-PCR 24 h post-transfection.

### Lung histology

Excised lungs were stored in Safefix II (Fischer Scientific, Pittsburgh, PA) for 48 h for fixation and then transferred to 70% ethanol until paraffin-embedding for H&E staining. For staining of OxPhos, lung sections were stained with antibody against oxidized phospholipids (clone EO6, Cat# 330001S from Avanti Polar Lipids Inc. used at 1:100 dilution) in combination with Vector M.O.M immunodetection kit (Vector Laboratories, Cat# PK-2200). DAB peroxidase substrate was used to detect positive EO6 staining (brown colour). The slides were then counterstained with hematoxylin, dehydrated, cleared and mounted.

### Assay of secreted cytokines

Lungs were homogenized in a buffer containing 50 mM Tris·HCl, pH 7.4, 150 mM NaCl, 0.02% Tween 20, and Complete Mini, EDTA-free protease inhibitor (Roche Applied Science) and debris-free supernatant was used for cytokine measurement. Secreted cytokines in mouse lung extracts were measured by magnetic bead-based assay (Bio-Rad Laboratories, Hercules, CA) according to the manufacturer's instructions. Data was acquired and analysed using Luminex automated system (BioRad Laboratories, Hercules, CA, USA). The level of IL-10 protein in supernatants of *ex vivo* cultures was measured using a DuoSet Elisa kit (R&D Systems, Cat # DY417) according to the manufacturer's protocol.

### Measurement of albumin in BAL

Amount of albumin leak in BAL was measured using mouse albumin-specific ELISA kit (Abcam, Cat # ab108792) following the manufacturer's instructions.

### MPO assay

MPO assay was performed as described previously^16^. Briefly, a 2.5 mg ml^−1^ solution of O-Dianidinedihydrochloride (Cat # D3252) (Sigma, USA) in 0.1%BSA/PBS was mixed with another solution of 0.1% H_2_O_2_ prepared in 0.1%BSA/PBS. Two hundred microlitre of this solution was added to 50 μl of standard or sample (BAL) and incubated for 15 min at 37 °C. The reaction was stopped by adding 50 μl of 2% sodium azide (Sigma-Aldrich, USA). The colour developed was read at 450 nm wavelength of light.

### Flow cytometry

Single-cell suspensions stained with combinations of antibodies listed above were acquired on FACS Aria (BD Immunocytometry Systems) and the data were analysed using FlowJo software (Tree Star).

### Site-directed mutagenesis

K107 and K395 SUMOylation sites in PPARγ were mutated to arginine using QuikChange II XL Site-Directed Mutagenesis Kit (Cat # 200521) from Agilent Technologies following manufacturer's instructions. Briefly, PPARγ overexpression construct (purchased from Addgene; Cat # 8895) was used as template in a PCR containing the primers harboring the desired mutations. Of note, the expression construct encodes PPARγ2, which is an isoform of PPARγ1, with the former containing 30 additional amino acids in the N-terminus in the human protein. PPARγ1 is expressed in many cell types including hematopoietic cells while PPARγ2 is expressed in adipose tissue although expression can be induced in other tissues by high-fat diet^57^. Importantly, the two proteins PPARγ1 and PPARγ2 have similar functions in transfection experiments^67^. Following PCR amplification, the methylated template strands were removed by digestion with DpnI and the undigested constructs containing the desired mutations were used for transforming transformation-competent *E. coli* cells. The plasmids were purified and used for transfecting RAW 264.7 cells (ATCC, Cat # ATCC TIB-71).

### Statistical analysis

Results shown are mean values±s.d. One-way ANOVA with Bonferroni *post hoc* test was used for multiple pairwise comparisons. Student's unpaired two-tailed *t* test was used for comparisons involving two groups. Differences between groups were considered significant when *P*≤0.05. Kaplan–Meier survival curves are derived from two experiments. The overall *P* value was calculated using log-rank test. All statistical analyses were performed using GraphPad Prism 5 software (La Jolla, CA, USA).

### Data availability

The data supporting the conclusions of the study are available from the authors upon reasonable request.

## Additional information

**How to cite this article:** Chakraborty, K. *et al*. The mito-DAMP cardiolipin blocks IL-10 production causing persistent inflammation during bacterial pneumonia. *Nat. Commun.* **8,** 13944 doi: 10.1038/ncomms13944 (2017).

**Publisher's note**: Springer Nature remains neutral with regard to jurisdictional claims in published maps and institutional affiliations.

## Supplementary Material

Supplementary Information Supplementary Figures
