# Supplementary material for: The mito-DAMP cardiolipin blocks IL-10 production causing persistent inflammation during bacterial pneumonia
Source: Nat Commun. 2017 Jan 11;8:13944. doi: 10.1038/ncomms13944 (PMC5241690; doi:10.1038/ncomms13944)
Supplement: Supplementary Information — Supplementary Figures [file ncomms13944-s1.pdf]

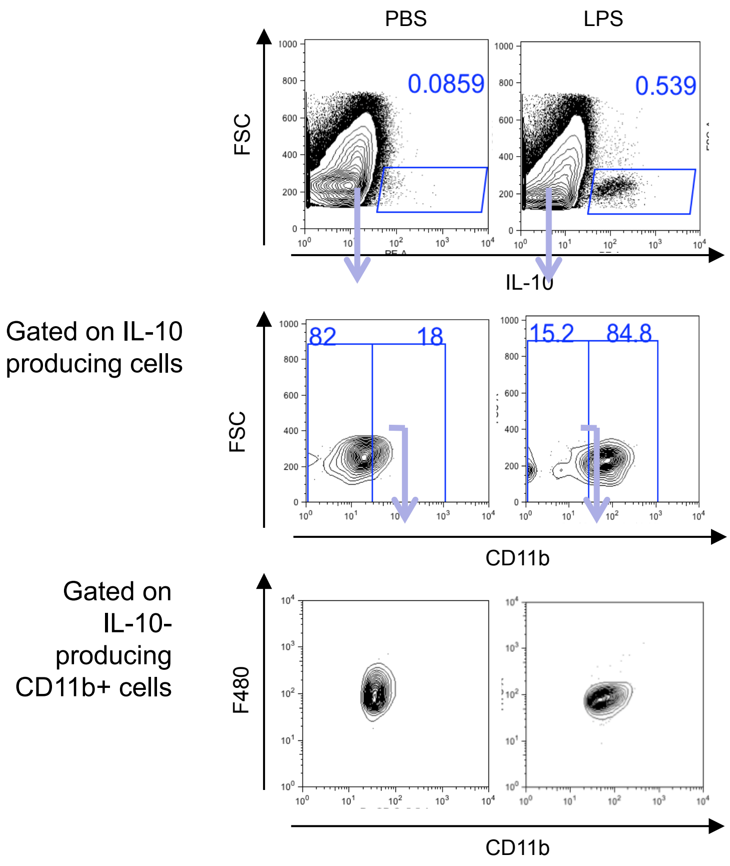

Supplementary Figure 1. Identification of IL-10-producing cells in the lungs of mice after LPS treatment. LPS (15 µg/dose) or PBS was instilled intratracheally into mice once for three consecutive days. 24 h post-treatment, IL-10 producing cells in the lung were analyzed by flow cytometry using gating strategies shown. Data shown are representative of two independent experiments (n=3 mice per group).

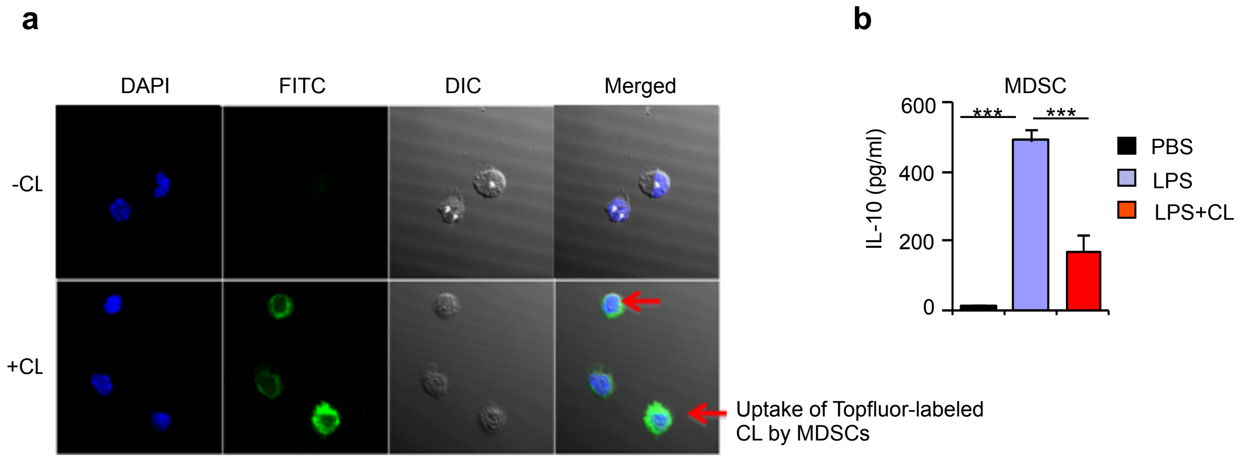

Supplementary Figure 2. CL uptake by lung MDSC cells and inhibition by cardiolipin (CL) of LPS-induced IL-10 production from the MDSCs. (**a**) Uptake of Topfluor-labeled cardiolipin by lung MDSCs as assessed by confocal microscopy. Cell nuclei were stained with DAPI. (**b**) CD11b $^{+}$Ly6G^{int}F4/80^{+} lung MDSCs were flow-sorted and cultured *ex vivo* for 6 h with LPS (1µg/ml) either alone or in combination with cardiolipin (10 µg/ml). Secreted IL-10 in the culture supernatant was measured by ELISA. Data presented are mean ±s.d. *** P <0.001.

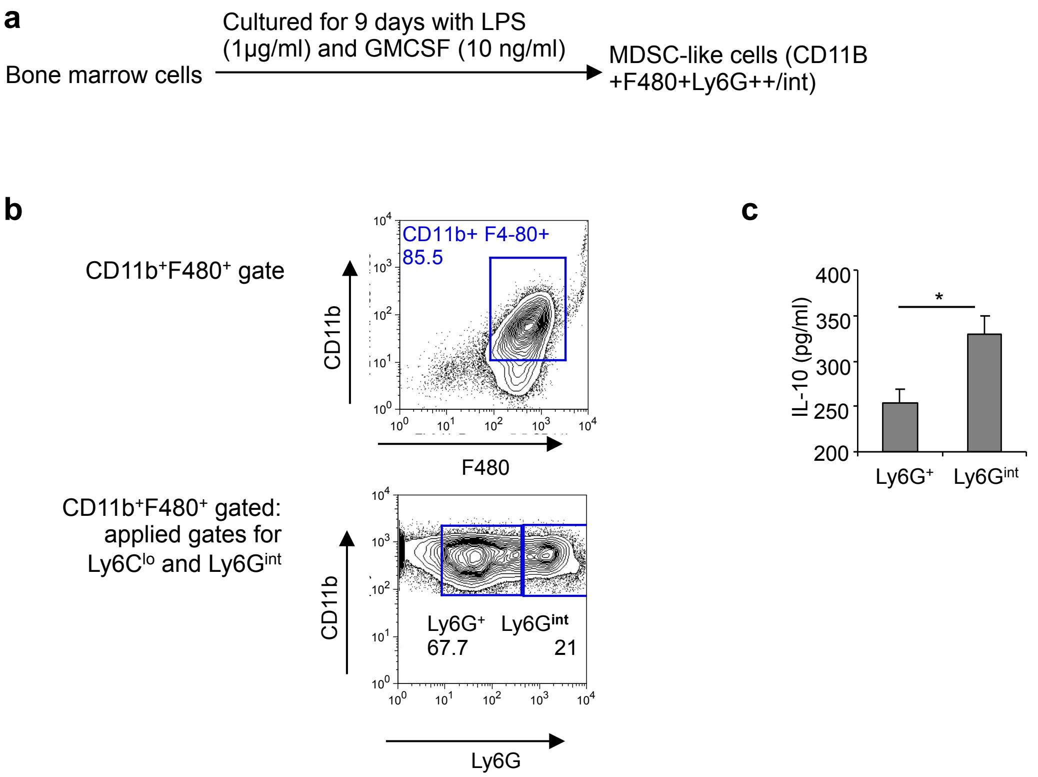

Supplementary Figure 3. Generation of bone-marrow-derived MDSCs for adoptive transfer into mice. (**a**) Schematic of generation of bone marrow-derived MDSCs. (**b**) Flow cytometric analysis of bone marrow-derived MDSC-like cells. (**c**) Secreted IL-10 from bone marrow-derived MDSC-like cells following 6 h LPS (1 µg/ml) stimulation as measured by ELISA. Data presented are mean ± s.d. * P <0.01.

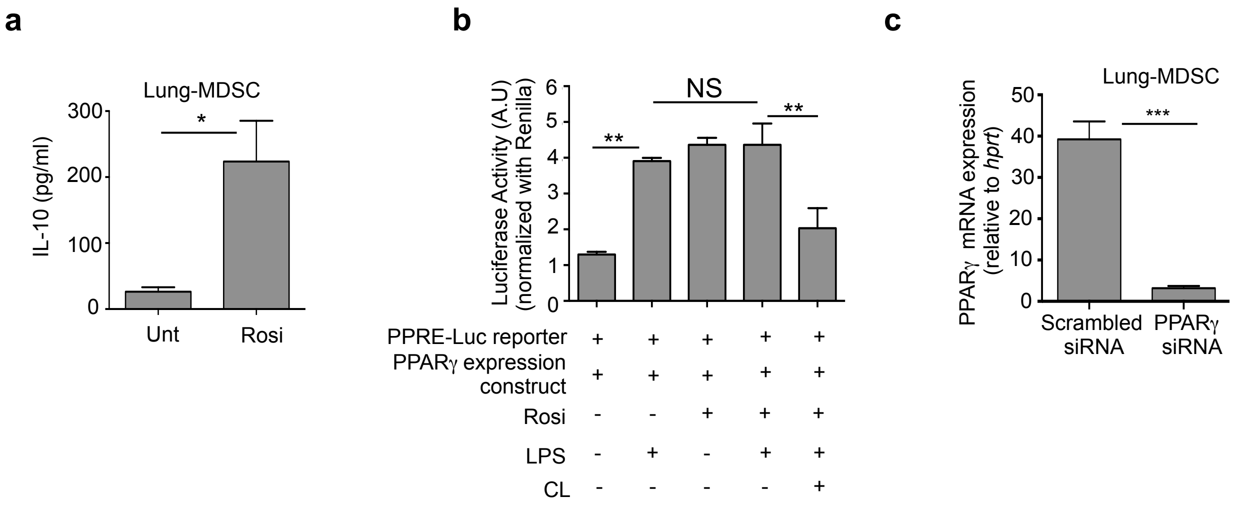

Supplementary Figure 4. PPARγ regulates IL-10 gene expression in lung MDSCs. (**a**) Lung MDSCs were treated *ex vivo* with or without Rosiglitazone (Rosi-10 μM) for 6 h. Secreted IL-10 in the culture supernatant was measured by ELISA. (**b**) RAW 264.7 cells were transfected with both PPRE-Luciferase reporter construct and PPARγ expression vector. 24 h post-transfection, cells were stimulated with Rosi or LPS, either alone or in combinations with cardiolipin. 2 h post-stimulation, reporter activity was measured. (**c**) Lung MDSCs were isolated from LPS-treated mice and transfected (1 X 10^6 cells /condition) with scrambled or *pparg*-targeted siRNA (10 nM). *pparg*-gene expression was studied by qRT-PCR. Data shown are mean ± s.d. and all data are representative of two independent experiments. *P≤0.05,**P≤0.01, ***P≤0.001.

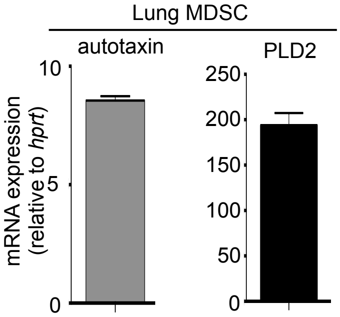

Supplementary Figure 5. Relative mRNA expression of autotaxin and PLD2 in lung MDSCs. Data shown are mean ± s.d. and representative of two independent experiments.

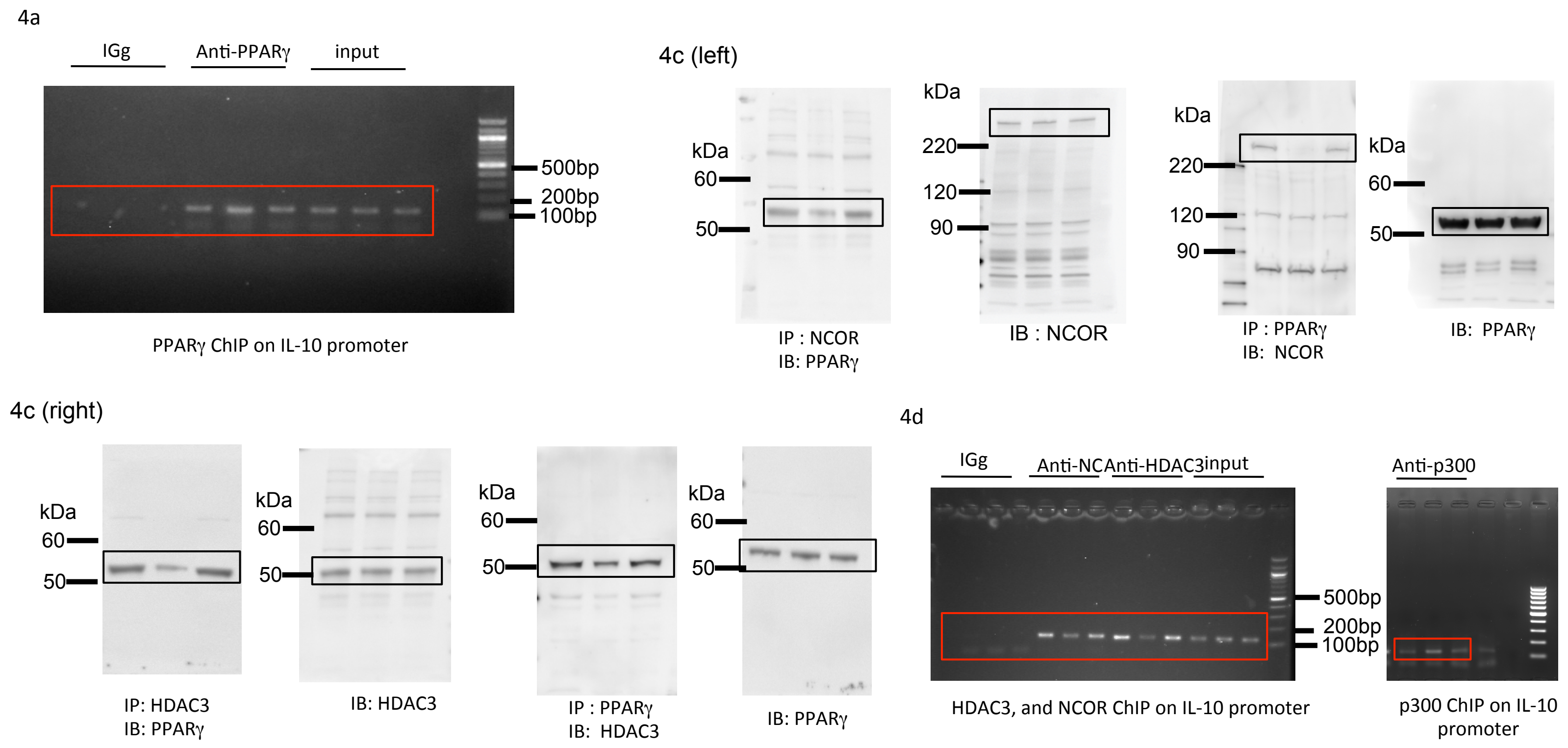

Supplementary Figure 6. Complete scan of Western blot data shown in Figure 4.

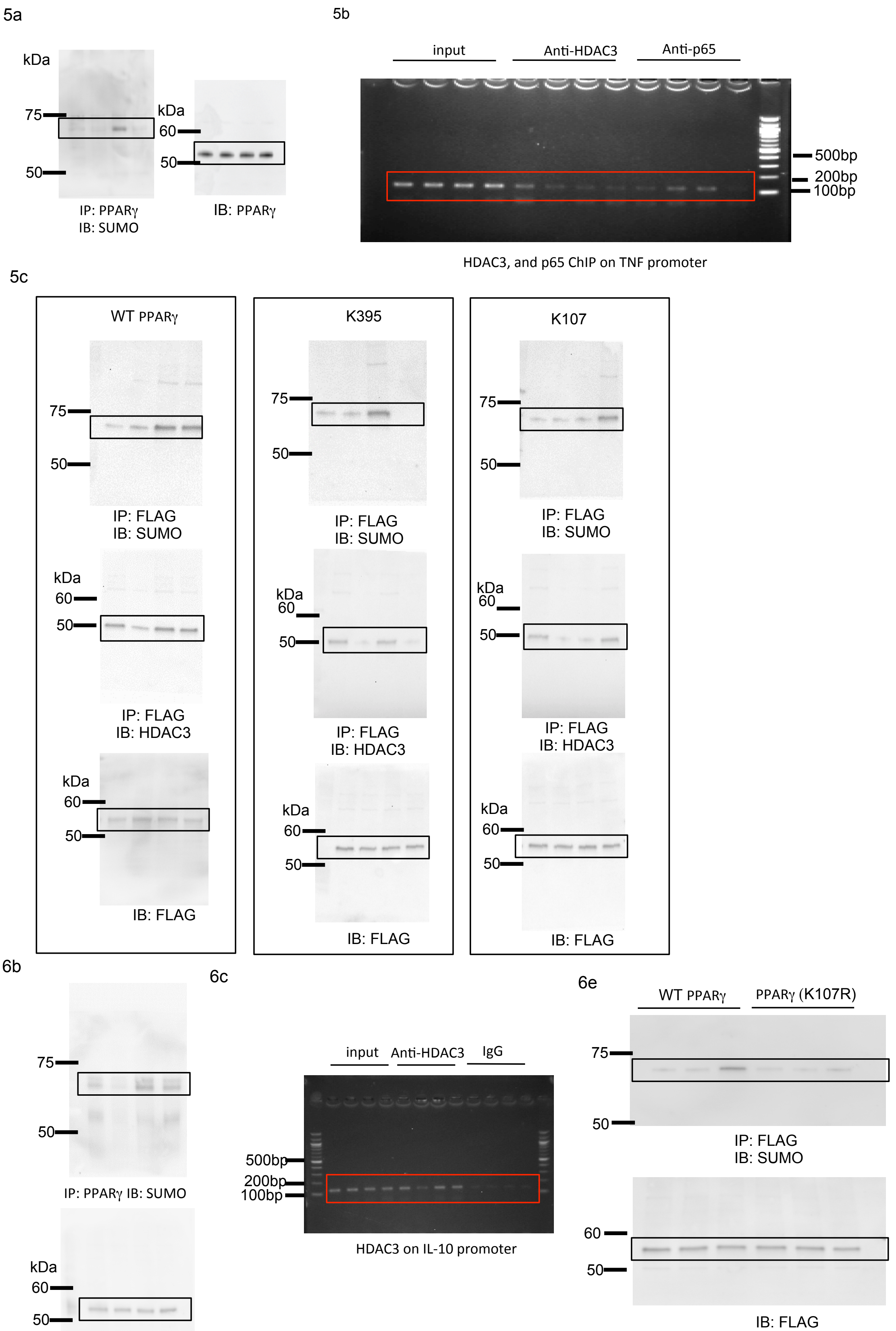

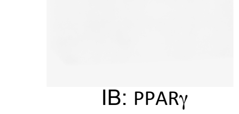

Supplementary Figure 7. Complete scan of Western blot data shown in Figures 5 and 6.

7f

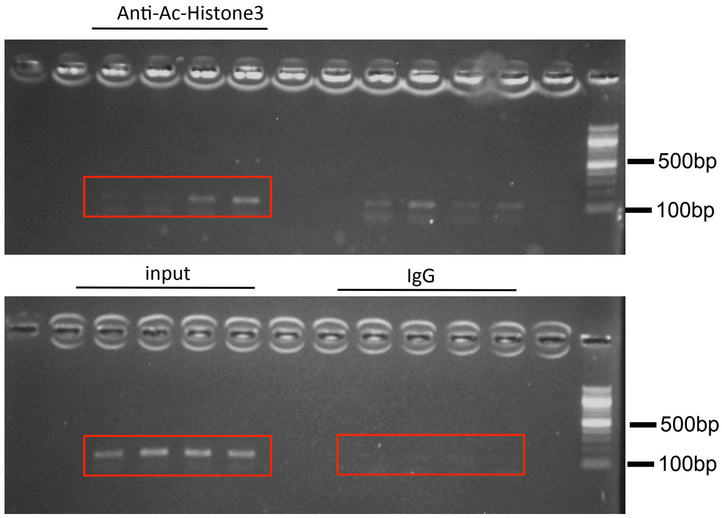

8a

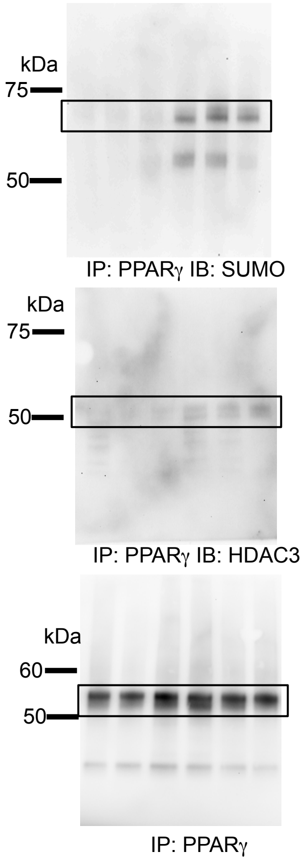

8b

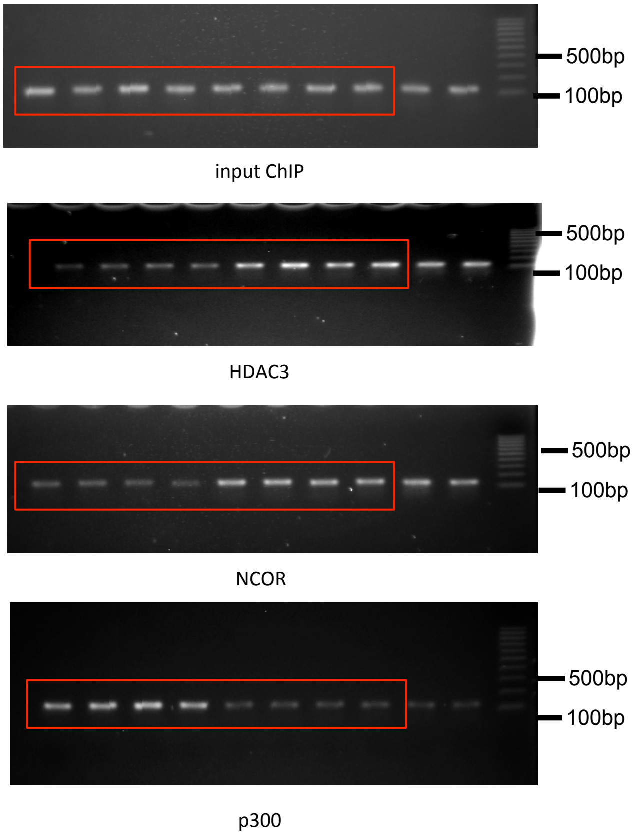

Supplementary Figure 8. Complete scan of Western blot data shown in Figures 7 and 8.
